# Divergent Importance of Chronobiological Considerations in High- and Low-dose Melatonin Therapies

**DOI:** 10.3390/diseases9010018

**Published:** 2021-03-09

**Authors:** Rüdiger Hardeland

**Affiliations:** Johann Friedrich Blumenbach Institute of Zoology and Anthropology, University of Göttingen, 37073 Göttingen, Germany; rhardel@gwdg.de

**Keywords:** circadian, entrainment, inflammation, melatonin, mitochondria, receptor saturation

## Abstract

Melatonin has been used preclinically and clinically for different purposes. Some applications are related to readjustment of circadian oscillators, others use doses that exceed the saturation of melatonin receptors MT_1_ and MT_2_ and are unsuitable for chronobiological purposes. Conditions are outlined for appropriately applying melatonin as a chronobiotic or for protective actions at elevated levels. Circadian readjustments require doses in the lower mg range, according to receptor affinities. However, this needs consideration of the phase response curve, which contains a silent zone, a delay part, a transition point and an advance part. Notably, the dim light melatonin onset (DLMO) is found in the silent zone. In this specific phase, melatonin can induce sleep onset, but does not shift the circadian master clock. Although sleep onset is also under circadian control, sleep and circadian susceptibility are dissociated at this point. Other limits of soporific effects concern dose, duration of action and poor individual responses. The use of high melatonin doses, up to several hundred mg, for purposes of antioxidative and anti-inflammatory protection, especially in sepsis and viral diseases, have to be seen in the context of melatonin’s tissue levels, its formation in mitochondria, and detoxification of free radicals.

## 1. Introduction

Melatonin has been tested as a protective drug against numerous diseases and disorders, both preclinically and clinically. The applications and preclinical experiments were only partially oriented at the well-documented physiological roles of melatonin as a chronobiotic. In fact, the majority of studies had focused on a different field, antioxidative protection, using often considerably higher doses than applicable in a meaningful way for chronobiological purposes. Although cross-connections between chronobiological and antioxidant aspects have sometimes become apparent [1,2], the suitability of different doses has remained to be a matter of concern and confusion. 

The chronobiological role of melatonin is since long established. The main secretory source of melatonin is, in mammals, birds and various other but not all vertebrates, the pineal gland, which synthesizes and releases this compound in a circadian fashion, with a prominent maximum at night [3,4,5,6], Additional chronobiological functions are known in seasonality. However, this is preferentially relevant to seasonal breeders, but only of marginal importance to the human [7]. In mammals, the predominantly nocturnal secretion of melatonin is steered by the circadian master clock, the suprachiasmatic nucleus (SCN), and transmitted to the pineal gland via a neuronal connection [5,6,8]. Additionally, melatonin formation is modulated by input pathways to the SCN [9,10] and to the pineal gland [11]. From this gland, melatonin is released into both the circulation and, via the pineal recess, the third ventricle of the brain, in even higher concentrations [12,13,14]. For decades, the role of melatonin had been predominantly seen in the transmission of the signal “darkness” to numerous, if not all organs [5,15,16], thereby correcting undesired deviations within the circadian multioscillator system and contributing to both the internal synchronization of the manifold circadian rhythms and their entrainment with external time cues [17,18]. This also implicated direct effects of melatonin on the SCN, leading to circadian phase shifting, also known as the chronobiotic role of this compound [19,20,21,22]. Circulating melatonin had been assumed to be the major source of chronobiotic effects, but more recent evidence indicates that melatonin release into the third ventricle, from where this agent gets direct access the adjacent SCN, may be even more important [23,24]. 

These chronobiological considerations have, however, disregarded an important fact, namely, that melatonin is not exclusively formed in the pineal gland, but also in other, presumably all organs [25,26]. The amounts present in extrapineal tissues are not at all negligible, but rather exceed the quantities in pineal gland and circulation by orders of magnitude [25,27,28,29]. Although this is well-known since long, this point is missed by many researchers. Hence, one can frequently read the false statement that “the pineal gland is the main site of melatonin synthesis”. In fact, the pineal gland is only the main source of circulating melatonin, whereas tissue melatonin is, under normal conditions, only poorly released [25,30]. 

The fact that melatonin is synthesized in many places within the body, at much higher quantities than in the pineal gland, sheds light on the necessity of defining melatonin’s physiological role in a way exceeding the chronobiotic aspects. This is the more important as circadian variations of tissue melatonin are either much smaller than in those in pineal and circulation or even almost absent [25,30,31]. In terms of treating humans with melatonin, this opens another area of application beyond chronobiology. In particular, this is valid for the field of antioxidative protection by exogenously administered melatonin. As will be discussed in detail, much higher doses have been applied for preventing oxidative damage. Such treatments, which had been regarded for years as being extremely unphysiological, can now be seen in a different, more acceptable way. Nevertheless, the pharmacological administration of high-dosed melatonin for counteracting oxidative damage may be expected to interfere with the orchestration of circadian rhythms and, therefore, to cause undesired problems in the functioning of the body, Under this perspective, the discussion of doses and the weighing of justified high doses to prevent tissue damage vs. chronobiological demands seem to be an undeniable necessity.

## 2. Basis of Chronobiotic Treatment: Receptor Affinity, Pharmacokinetics, and Phase Response Curve

In mammals, two G protein-coupled receptors (GPCRs), MT_1_ and MT_2_ [32,33], are decisive for most of the known receptor-mediated actions of melatonin. Details of their expression, modulation and downstream pathways have been published elsewhere [25,34,35,36]. Generally, the affinity of a receptor has to correspond to the physiological levels of the ligand, at best with a half-saturation in the range of moderately elevated ligand concentrations, to allow both up and downregulation according to ligand variations. As far as melatonin is transported to target cells via the blood stream, this means that half-saturation should be lower than maximum levels in blood plasma (≤1 nM). In fact, the pKi values for human MT_1_ and MT_2_ have been mostly found to be in the range of 10 and 9.5, respectively, with some differences between studies [37,38]. Therefore, resetting of circadian rhythms and circadian-related functions such as sleep requires blood plasma levels of melatonin in the range of 0.1–1 nM. For calculating doses, one has also to consider bioavailability, which is restricted by uptake (depending on administration route), metabolic destruction (especially elimination by hepatic first-pass metabolism), and distribution within the body. As a result of metabolic destruction and excretion, the half-life of normally dosed, orally administered immediate-release melatonin (1–3 mg) is in the circulation rather short and mostly found in the range of 20–30, sometimes 45 min [39,40]. However, clearance values depend on dose and also on route of administration. Longer half-lives were reported when the doses were elevated to 10 mg [41] or when other routes such as rectal or vaginal administration [42] were tested. To extend the presence of melatonin in the circulation, it is, of course, possible to use higher doses, however, at the expense of transient receptor oversaturation. Whether this is relevant or not, has been a matter of debate. As discussed elsewhere [36], oversaturated GPCRs can be internalized upon C-terminal modification and β-arrestin binding. Although internalization was demonstrated in a few cases for MT_2_, the evidence for this had remained rather weak in the case of MT_1_ [36].

MT_1_/MT_2_-independent actions through other binding sites are, in principle, also possible, although their relevance is poorly explored in the medical context. Moreover, several of such actions that have been frequently cited remain uncertain or have been dismissed. A protein previously discussed as a “melatonin receptor MT3” has been identified as the enzyme NRH:quinone oxidoreductase 2 (NQO2; alias quinone reductase 2, QR2) [43,44,45]. However, NQO2 is not activated but rather inhibited by melatonin, does not initiate any known signal transduction pathway, and is not sufficiently specific for melatonin. Therefore, it does not meet the required criteria for a receptor [25]. Transcription factors such as retinoid orphan receptors (RORs), in particular, RORα, but also other splice variants and homologs, had been considered as nuclear melatonin receptors in numerous publications, but had turned out to not bind melatonin at reasonable concentrations [46,47,48]. Melatonin binding was also reported for calreticulin and two nuclear proteins, one of which with homology to calreticulin [49], and also for tubulin [50,51], but there is to date no evidence for a relationship to chronobiotic effects. This reservation may be also valid for calmodulin (CaM) as another, more thoroughly investigated binding site [52]. However, its relevance has been debated in terms of affinity [32]. Although the binding to CaM alone may be insufficient for a physiological role, its affinity seems to be considerably elevated when conformationally changed by association with Ca^2+^/CaM-activatable enzymes [53]. Although CaM-mediated effects of melatonin may participate in circadian changes of various cell functions, no evidence exists for a role in phase resetting of circadian oscillators.

**Figure 1:**
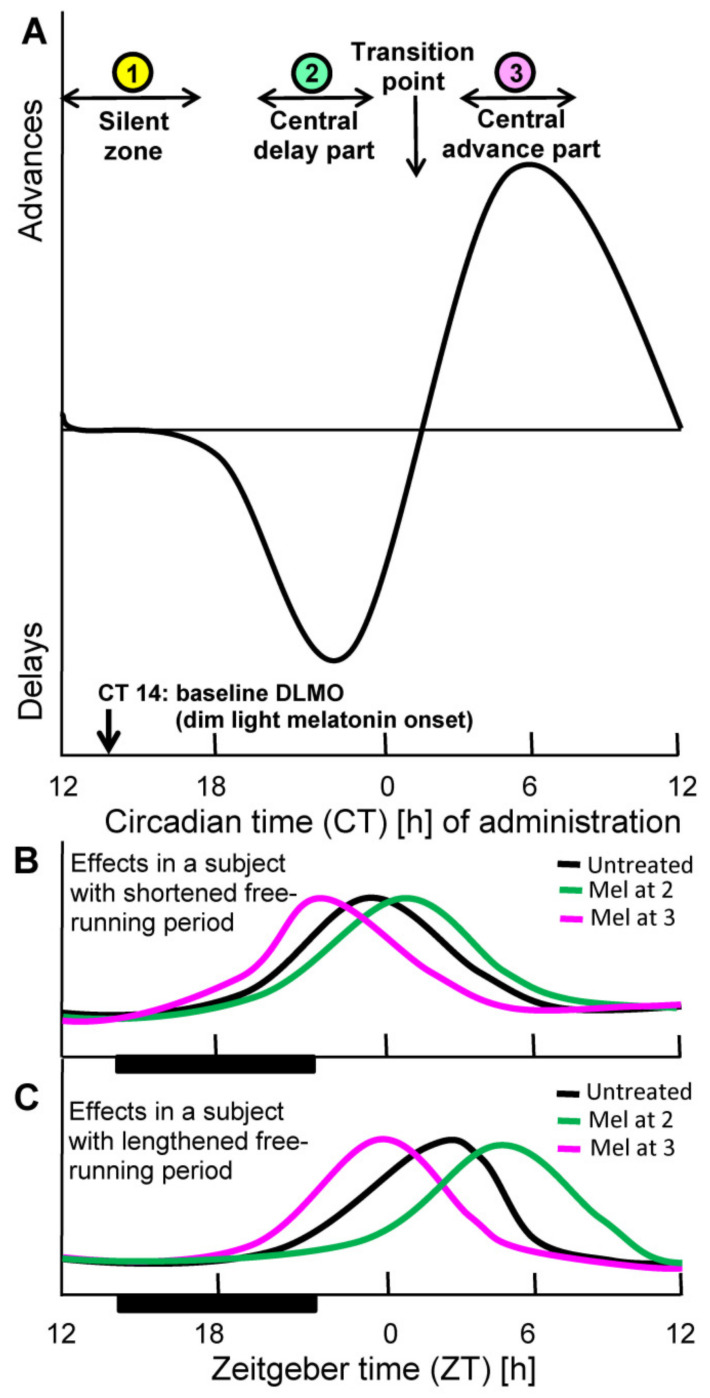
Phase resetting by melatonin according to the Phase Response Curve (PRC). (**A**): Schematic depiction of human PRC for low doses of immediate-release melatonin. The curve is based on data by Lewy et al., obtained with 0.25 mg melatonin [26]. Original data have been omitted for improving comprehensibility of the principles. Notably, the extent of phase shifts (here: range of a very few hours) and some other details of such PRC curves are also dose-dependent. However, higher doses such as 10 mg do not necessarily increase, but may rather reduce the amplitude of the PRC [61]. PRCs are determined under free-running conditions, in which the phases of the spontaneous period are referred to circadian time (CT) (one free-running cycle = 24 subjective hours). The influences of resetting capacity are also detectable under entrained conditions and become evident by changes in the phase positions of prominent features of circadian rhythms, such as maxima. (**B**,**C**): Predicted effects of melatonin in different phases of the PRC, in subjects with shortened (**B**) or lengthened (**C**) spontaneous periods, however, under synchronized conditions in a light-dark cycle (black bars: darkness). Under synchronized conditions, time is standardized as Zeitgeber time (ZT); in a 12:12 h light/dark cycle, ZT 0 corresponds to light onset; under deviating light/dark cycles, ZT is adjusted to the middle of dark phase. For facilitating comprehensibility, a notional rhythm with a maximum in the morning has been depicted. A rhythm of an oscillator gene in the SCN would be preferable, but these data are not available for the human. Rhythms of oscillator genes in other human cells or tissues have been reported, but preclinical studies have shown that their phasing deviates between tissues, sometimes strongly, and are, therefore, not suitable.

Another point of fundamental relevance to chronobiotic efficacy concerns timing within the circadian cycle. Phase resetting by whatever time cue, including biochemical signals, depends on the time point of administration. This dependence is usually described as a phase response curve (PRC). This is insofar highly important as the degree and direction of phase change within the circadian period. The PRC typically contains delay and advance parts and, in between, silent zones in which no substantial changes are achieved. The PRC for melatonin has been determined in the human (Figure 1), for sighted and blind subjects [19,54,55,56]. When closely looking at the details of the PRC, one will state that the frequently read recommendation for melatonin intake about half an hour before bedtime can remain insufficient for resetting. Although a low dose of melatonin given at this time point is rather reliably capable of inducing sleep onset, this does not yet shift the circadian clock [22]. The circadian phase of dim light melatonin onset (DLMO), which is usually taken as a circadian reference point is typically found around circadian time 14 hours (CT14) [22], which is, however, within an extended silent zone, in which no phase shifts occur (Figure 1). The delay part of the PRC typically begins several hours later and reaches its maximal values around CT22. After a transition point around CT2 that reflects the change from delay to advance, the maximal advance is observed at CT6, i.e., in the early morning. The lesson from these details is to not confuse phase shift with sleep onset [22], although the latter is also under circadian control and although the SCN is also involved in sleep induction [57,58,59,60].

Notably, the form of the PRC is dose-dependent. With higher doses, the presence of elevated melatonin covers larger parts of the PRC and integrates sections of different functionality. For example, a high dose given at bedtime, which is not cleared within a few hours, can induce phase delays, if elevated blood concentrations extend into the delay part. However, a more appropriately timed administration that hits the circadian oscillators of the SCN in the middle of the delay phase, would be more efficient. The problem of integration of different phases within the SCN leads, in fact, to a lower entrainment capacity of higher melatonin doses than of lower ones [54,62]. In the context of synchronization, one should also take into account that the form of an entraining signal is also relevant to its efficacy. As outlined elsewhere [22], circadian oscillators are more sensitive to so-called nonparametric signals characterized by a rapid increase are more effective than parametric ones that describe the relationship to the permanent height of the signal. In the case of melatonin, the parametric measure would be the finally attained concentration. Therefore, the effect of an increase that may be moderate in extent but rather rapid can be more efficient than a slow increase toward a higher level. Thus, the effect of melatonin given around DLMO that extends because of dose into the delay part can be expected to be poorer than a lower but more appropriately timed dose given shortly before or at the beginning of the delay part of the PRC.

## 3. Tissue and Organellar Melatonin

Tissue concentrations of melatonin have been determined in a number of cases [26,27,28,29,30], but not yet throughout all organs. Nevertheless, the existing data clearly show that tissue melatonin is of fundamental importance. Instead of summarizing all available data for the different parts of the body, the important consequences of this insight shall be addressed. A particular aspect that has gained relevance in the course of the last years concerns melatonin synthesis in mitochondria and the resulting levels in these organelles as well as in the cytoplasm. Mitochondrial formation of melatonin, first suggested as a cell biologically founded hypothesis [63], was first demonstrated in murine oocytes [64] and, in more detail, in the rat brain [31], followed by further evidence concerning the choroid plexus [65] and corresponding assumptions for the skin [66]. For more details of melatonin-mitochondrial relationships and similar findings in the botanical field see ref. [67]. Notably, melatonin concentrations have been found to be higher in mitochondria than in any other compartment of vertebrate cells [67,68]. In addition to its intramitochondrial synthesis, melatonin can also accumulate in these organelles when administered exogenously, e.g., by intravenous infusion. This has been convincingly shown in freely moving rats, when melatonin was continually infused during the day to attain night-time levels and its distribution was followed by a [^3^H]–labeled fraction [61]. Of note, mitochondrial melatonin formation has gained recent actuality concerning the hijacking of mitochondria by viruses including SARS-CoV2 [69].

The relevance of tissue melatonin, its poor release from most organs and mitochondrial-cytosolic concentration gradients now forces investigators to dismiss a frequently forwarded earlier assumption, namely, that of the freely diffusible molecule which easily crosses all membranes. This assumption was based on melatonin’s amphiphilicity and data obtained in artificial lipid bilayers [70,71]. However, the low rates of release from most tissues raised the question of whether melatonin really enters cells with ease, although it is hindered to leave them [30]. If this were not due to melatonin sequestration by nonreceptor proteins, the nearly free diffusion had to be wrong. In fact, it has meanwhile been shown that melatonin uptake through the plasma membrane is facilitated by transporters of the GLU/SLC2A (solute carrier 2A) type [72,73]. As SLC2A represents a superfamily, a more specific subtype characterization would be desirable, especially with regard to the understanding of selectivity, since SLC2A proteins are otherwise known as transporters of glucose, i.e., a compound that might outcompete melatonin because of its considerably higher blood plasma concentration. Therefore, the SLC2A subform involved in melatonin uptake has presumably a lower affinity for glucose. At least, the participation of an SLC2A protein, which is typically a uniporter, would explain why melatonin can be imported, in spite of being poorly released. The uptake of melatonin from the cytosol to mitochondria has been ascribed to PEPT1/2 transporters [74], which would, again, explain a widely unidirectional transport, since their actions are proton-driven.

Collectively, the findings on high melatonin levels in mitochondria raise the question of intracellular receptors. In fact, the presence of MT_1_ in the outer mitochondrial membrane of neurons was reported [31]. In gastric endothelial cells, both MT_1_ and MT_2_ were found to be present in mitochondrial membranes [75]. If mitochondrially located melatonin receptors have the same affinities as those present in the plasma membrane and are not altered in this regard by modification, this would mean that regulation by melatonin has to take place in the same concentration range as in the circulation. As far as melatonin enters the receptors from the cytosol or from the intermembrane space [76], this may not be a problem, as long as melatonin is not released in too high amounts from the mitochondrial matrix, its site of synthesis where the required coenzymes are present. However, if concentrations rise above receptor saturation, e.g., because of a high-dose melatonin treatment, the effects via MT_1_ and/or MT_2_ cannot exceed those by levels that are close to saturation. However, numerous studies have used doses that should have exceeded by far the saturating concentration. For many years, such approaches have appeared to a number of researchers as questionable, because they seemed to be beyond the physiological regulation range. 

However, these judgments may have been precocious, since melatonin displays important additional properties. For instance, it interacts with the mitochondrial transition permeability pore (mtPTP), causing inhibition and reduction of opening time [77,78,79], effects that require higher concentrations (somewhat below µM levels [77]) than receptor saturation. Moreover, it directly interacts with reactive oxygen species [80,81,82,83]. Of course, antioxidative protection by melatonin is not restricted to free radical scavenging, but also comprises various other effects that are receptor-mediated [25,84,85]. However, the property as a scavenger allows increases in protective efficacy by orders of magnitude above receptor saturation. In fact, antioxidative protection against neuronal damage by cerebral ischemia was documented to be possible in MT_1_/MT_2_ double knockout mice [86]. More data of this kind would be desired for supporting the efficacy and usefulness of high melatonin.

## 4. Contrasting Judgments on the Tolerability of Melatonin by Agencies and Researchers

In the minds of many researchers and clinicians, melatonin appears to be conceived as an agent that requires particular caution. Several reasons for this may have contributed to this opinion. First, the low levels of circulating melatonin seem to indicate that one should not exceed these levels by exogenous administration. However, the high amounts of tissue melatonin are disregarded when considering the blood levels as the only physiological measure. Second, melatonin’s previous investigational use for interfering with reproductive biology, including that of a contraceptive [87,88], has apparently become a matter of concern. Although melatonin effectively controls reproduction in seasonal breeders [89,90,91], a comparable relevance to the human as a nonseasonal breeder has not been detected, despite some effects on prolactin secretion [92]. Its contraceptive activity has largely melted down to an assumed synergism with a synthetic progestin, norethisterone [87]. Third, long-term melatonin treatment had been reported to decrease sperm counts in human males, but this was only observed in a very few individuals [93]. Collectively, such reservations have contributed to an overcautious consideration of melatonin for treatments, e.g., in the context of sleep improvements. For instance, a prolonged release preparation was only approved for the use in insomnia patients of 55 years and over, at a dose of 2 mg, a decision that strongly contrasts with much higher approved doses for synthetic melatonergic agonists, such as ramelteon (8 mg) or agomelatine (25 or 50 mg), although these compounds show higher or similar receptor affinities [60,94], and also despite concerns of toxicity, especially in the case of agomelatine [95]. Of course, very moderate doses of melatonin, even below 1 mg, are sufficient for reducing sleep onset latency [22,96], but approved doses such as 2 or 3 mg are insufficient for sleep maintenance throughout the night. Higher doses such as 50 or 100 mg have been suggested for improving sleep maintenance [97]. The dose of 50 mg has occasionally been used, with some success [98]. 

While only low doses have been accepted for treatments of circadian regulation, considerably higher amounts have been used for purposes of protection (cf. Section 5). In fact, strongly elevated levels of melatonin that exceeded physiological concentrations were required for achieving sufficient protection. The remarkable tolerability of melatonin was demonstrated in a study in ALS patients, who were treated with suppositories of 300 mg melatonin daily for up to two years [99]. In terms of bioavailability, this dose should be higher than the same amount when administered orally, since the rectal application reduces the losses by first-pass metabolism [100,101]. An even higher dose of 1 g/d was administered to dermatological and endocrinological patients for one month, without severe side effects [102]. Similarly, high doses were applied in various studies that demonstrated successful treatments of viral diseases of animals and of sepsis in neonates, as summarized elsewhere [100]. According to dose translation, the recommendation for adult humans of 75 kg amounted to 600 mg/day, to be given orally in up to five partial doses [100]. This dose was suggested for counteracting the inflammatory cytokine storm in COVID-19 with its resulting coagulopathies and mitochondrial impairments [100]. Other papers also recommended elevated doses, such as 100–400 mg [103] or about 600 mg [104]. In a study on liver resection, the safety margin of melatonin was determined to be as high as 3750 mg/d for a person of 75 kg [105]. Collectively, these data clearly demonstrate that by orders of magnitude higher doses of melatonin are possible and acceptable than those approved for the treatment of circadian disorders, sleep difficulties and depression, at least under life-threatening conditions in which rescuing of a patient is by far more important than the possibility of a transient circadian disturbance or endocrine imbalance.

## 5. The Rationale for Chronobiotic and Nonchronobiotic Treatments

Although melatonin is just a single compound that exerts numerous effects, it would be a mistake to equate all its actions in the various fields. This opinion does not primarily refer to its pleiotropy toward different organs [25], but rather to functional fields, in particular, circadian regulation, soporific effects, and cell protection. Although there are undoubtedly cross-connections between these fields, it seems important to distinguish between them with regard to doses and timing. 

Sleep, including sleep induction, is controlled by the circadian system, however, not exclusively. In particular, the homeostatic drive to sleep as a consequence of insufficient sleep duration or quality [96,106,107] and immunological sleep induction in the context of infections have to be considered [108,109]. Of course, these different influences can overlap and even mutually interfere [107,110,111]. Moreover, melatonin is also involved in both circadian and immunological regulation [25,110,111,112,113]. However, melatonin’s actions on sleep are not generally mediated via the circadian system. Although the opening of the so-called sleep gate is under circadian control, sleep induction by melatonin is not necessarily associated with phase shifts [22]. As a consequence of these considerations, effects on the circadian master clock and on functions directly controlled by the SCN can, at first glance, be achieved by low doses of melatonin around or somewhat below receptor saturation, i.e., 1–3 mg orally. This might be judged in the same way for other MT_1_- or MT_2_-mediated functions. In particular, experience has shown that the shortening of sleep onset latency only requires such low doses, even below 1 mg [96].

However, these conclusions find some practical limits. One of them concerns the short half-life of melatonin in the circulation. If longer-lasting effects are desired, short-acting formulations may not suffice. Even prolonged-release pills may not be fully satisfactory [60]. The second limit results from frequently overlooked properties of the PRC. First, melatonin is poorly phase-shifting around DLMO [19,22]. Therefore, phase shifts require elevated melatonin in the delay or advance parts of the PRC, as outlined in Section 2. Second, the extent of phase shifts achieved by melatonin in humans is rather low and remains in the range of a very few hours, under normal conditions. However, it is important to distinguish between conditions under which a phase shift is desired (1) in an entrained person, or (2) in one who has been previously dysphased, e.g., by a transmeridian flight, or (3) in a nonentrained individual, e.g., a blind. 

Under condition (1), the small shifts that are possible by melatonin require a temporally appropriate application according to the PRC. If a short-acting formulation is applied around bedtime, the dose should be high enough to extend into the delay part. This may be particularly recommendable when the circadian deviation in the patient consists in a shortening of the free-running period (=spontaneous period). This situation is not uncommon in depressive disorders, especially of the bipolar type, in which the shortened period leads to misalignment [114]. A shortened free-running period results under entrained conditions to an advanced onset of activity and some other body functions. Expansion of the period leads, thus, to a normalization of phase positions of the respective circadian rhythms. At first glance, it may not appear to be evident why the shortening of an activity/rest cycle should be problematic. The explanation resides in the complexity of the circadian multioscillator system, which comprises numerous peripheral oscillators that differ in their coupling to the SCN [17,115] and, therefore, can dissociate and lead to internal desynchronization [17,114]. These forms of internal misalignment are believed to cause health problems [116,117]. However, circadian deviations of the period also exist because of elongations of the spontaneous period and likewise lead to internal misalignment and mood disorders. Therefore, a patient with bipolar disorder caused by an extended period requires period shortening, which can be achieved by advance shifts. These are possible by melatonin given in the advance part of the PRC at the end of the night [19,22]. In this case, a rather small dose of melatonin should suffice as there is no need to extend the action of melatonin, because the maximal advance usually coincides with the end of the night. Moreover, elevated melatonin would be undesired in the morning because of its sedating properties. Notably, melatonin, if appropriately timed, can cause both delay and advance shifts, whichever is required. Insofar, it is distinguished from a frequently prescribed antidepressant, lithium, which expands the spontaneous period and is, thus, suitable in cases of shortened periods, but counterproductive in patients with anyway lengthened periods [114]. It is therefore highly recommended to determine in bipolar patients circadian phase positions, which are indicative of deviations in period length, before treating these individuals with melatonin. This agent is not just a conventional antidepressant but requires consideration of its chronobiotic properties [114]. Importantly, a single phase shift will not suffice for a permanent correction of the deviating period, but requires repetition, which is no problem for a well-tolerated compound like melatonin.

Under condition (2), melatonin has been successfully used for reentraining rhythms [20,21,118,119,120]. However, experiences with treating jet-lag had remained rather diverse. To a certain extent, the negative results in a number of individuals were often related to the disregard of the chronobiological basis. First, the PRC for melatonin clearly shows that a large phase displacement by a transmeridian flight cannot be corrected by a single application of melatonin, which shifts the rhythm, at best, by a very few hours. Therefore, melatonin does not instantly correct the jet-lag, but can only accelerate the adaptation [118,119] by repeated applications. Second, a successful treatment against jet-lag requires consideration of the PRC to shift the internal rhythm into the desired direction. Third, the jet-lag is in part a consequence of dissociations within the multioscillator system. For instance, the activity/rest cycle can be easily uncoupled from the rhythm of core body temperature, an observation that had once led to the discovery of internal desynchronization [121]. The human activity/rest cycle exhibits a considerably broader spectrum of possible spontaneous periods (sometimes above 30 hours) than the temperature rhythm. As a consequence, the former is more easily entrainable than the latter. This author has personally experienced this difference when his activity rhythm was almost fully reentrained two days after a transmeridian flight, whereas the temperature maximum, which is normally found in the evening, remained in the morning for several more days. Although there is evidence for a synchronization of some oscillators within the multioscillator system by melatonin, there is no sufficient information on the specific velocities of entrainment, a gap that needs to be filled, as demanded elsewhere [17]. Finally, the light/dark patterns experienced in the course of the travel and thereafter under the new conditions also contribute to the outcome of adaptation. Generally, under conditions (1) and (2), the resetting or resynchronization by light should always be taken into consideration. This may also be used in combined treatments with melatonin and light, both to be applied in suitable phases according to respective PRCs and under consideration of light perception by melanopsin-expressing retinal ganglion cells [114]. 

Under condition (3), light-insensitive nonentrained blind people can, of course, be easily treated by melatonin in any desired phase that is suitable for resetting. The usefulness of melatonin has been demonstrated in several cases [54,55,62,118]. Again, low doses are sufficient and, as mentioned in Section 2, elevated doses such as 10 mg are less efficient than lower ones [62], presumably because of overlaps of different sections of the PRC. 

Low doses of melatonin are applicable for mainly two purposes, namely, (a) circadian resetting including mood therapies, and (b) facilitation of sleep onset. However, they are not promising for achieving full sleep continuation. This is, at least, obvious in individuals with substantial sleep difficulties, especially elderlies. Although some improvements of sleep duration or sleep quality can be obtained with conventional doses of melatonin, such as 2 or 3 mg, the aim of complete sleep maintenance has practically never been reached [60,122,123,124]. This statement is also valid for all synthetic melatonergic agonists, even though some of them such as ramelteon were somewhat more efficient in this regard than melatonin [60,69,125]. Higher doses such as 50 or 100 mg melatonin have been suggested for more efficient support of sleep maintenance [97], but these have not yet been applied in sufficiently large clinical studies. An alternative may possibly be a formulation with considerably extended release that approximately covers the entire night, especially for the use in patients with strongly reduced melatonin secretion. However, there may remain some skepticism even regarding this possibility. In the study by Weishaupt et al. [99], in which patients were treated for up to two years with daily 300 mg melatonin suppositories, which released the agent relatively slowly and caused a rather persistent elevation of blood levels, only about half of the subjects reported improvements of sleep continuation, whereas the others did not. In this context, it may be remarked that the treated persons were suffering of a severe disease. However, this should direct to a major, largely nonaddressed problem of the use of melatonin in insomnia studies, namely, the lack of identified reasons for the treatment-requiring sleep problems, which are especially observed in elderly and diseased subjects. These causes may be entirely different from circadian or pineal dysfunction and presumably have remained unidentified in many aged people, especially those with several comorbidities and polypharmacy. 

As mentioned in Section 4, melatonin can be used at much higher concentrations than required for circadian readjustments or for sleep onset facilitation. However, one cannot expect substantial circadian effects by these doses, as soon as long-persisting receptor oversaturation is caused by such a treatment. In the case of permanently enhanced melatonergic signaling, different sections of the PRC can be assumed to be affected that may become integrated and, thus, reduce or even annihilate a net phase shifting effect. However, consequences of permanent oversaturation have not yet been thoroughly studied as to whether or to what extent this may result in receptor desensitization in humans. On the other hand, actions by high melatonin that are observed in terms of sedation may include effects beyond MT_1_ and MT_2_ activation, such as inhibition of CaM-mediated nNOS activation by either melatonin or its metabolite *N*^1^-acetyl-5-methoxykynuramine (AMK) [126,127,128]. Moreover, some effects on ion channels may be possible, which, however, have been mostly attributed to MT_1_ or MT_2_ signaling, although numerous studies have required strongly oversaturating doses for obtaining such effects. With regard to the divergence of approaches and absence of dose-response considerations, a detailed discussion of this possibility would be premature.

Although long-lasting receptor oversaturation might be assumed to abolish melatonin’s effects on central and peripheral circadian oscillators, this does not mean that circadian oscillators are strongly affected by the absence of a melatonin signaling cycle. This may be especially valid for the SCN oscillators. Whether or not some changes cannot be fully excluded, the persistent cycling of circadian oscillators is highly likely. In this regard, the application of high or even extremely high doses of melatonin should not be taken as a caveat of severely disturbing the circadian clocks. Anyway, the very high doses of melatonin are usually applied for curing severe diseases or rescuing patients from life-threatening conditions. In such situations, circadian issues have to recede. 

Strongly elevated doses are especially applied for taking use of melatonin’s additional properties beyond of circadian regulation and GPCR signaling, such as binding to the mtPTP, which is half-saturated slightly below 1 µM [77], and free radical scavenging, which becomes effective at concentrations at which other antioxidants fail. Apart from its high affinity to the most destructive oxidizing free radicals, these considerations have to take into account two other remarkable properties of melatonin, first, that it forms in these reactions metabolites that do not initiate progressing prooxidative radical reaction chains [84,129] and, second, that it initiates a radical scavenger cascade. This cascade was first described for 4 consecutive reactions [130] and later extended to 10 detoxified radicals [131].

The relevance of these radical-detoxifying effects that require elevated concentrations also sheds light on the limits of efficacy of synthetic melatonergic agonists. None of them displays melatonin’s favorable properties of efficiently scavenging free radicals without initiating prooxidant reaction chains, a statement that also refers explicitly to its indolic analogs, whereas several nonindolic agonists can be concluded to be poor scavengers or generate prooxidant metabolites [60,95,125]. Moreover, no other agonist can be expected to display the extremely high tolerability of melatonin.

## 6. Conclusions

Melatonin can be used for different purposes, over a remarkably broad spectrum of doses. As far as actions depend on the GPCRs MT_1_ or MT_2_, low doses in the range of a few mg or even below 1 mg can be sufficient, according to receptor affinities that are half-saturated in the physiological range of circulating melatonin. This holds especially for the induction of sleep onset and for chronobiotic effects, such as phase shifting of circadian rhythms or modulation of period length. This is also relevant to the regulation of numerous other physiological functions that concern the majority of organs. Moreover, this comprises, e.g., the upregulation of antioxidant enzymes and the control of MT_1_/MT_2_-dependent immunological functions. Many of these physiological actions are not necessarily associated with changes in the circadian oscillator system, although these effects of melatonin are usually corresponding to rhythmic regulation, since melatonin, being an agent that cycles with high amplitude, transmits the signal “darkness” to the majority of organs. This consideration of fundamental importance is often overlooked. Its message is that an action on a rhythmic function cannot be equated with a change in a circadian oscillator.

This difference becomes particularly evident by a look at the PRC for melatonin. Phase shifts of oscillators are only possible in specific phases of application and vary with regard to both extent and, importantly, direction. In the silent zone of the PRC, melatonin does not substantially shift the oscillator, but may well transmit a message indicating darkness [22]. A most striking example for this is sleep onset induction, which works well in the silent zone. 

There are several physiological parameters that are sensitive to low doses of melatonin but are not sufficiently improved in cases of dysfunction. An example is the relatively poor efficacy of melatonin on sleep maintenance. To a certain degree, this may be related to the short half-life of melatonin in the circulation, as had been concluded by several researchers and had given rise to the production of long-acting melatonergic agonists and prolonged-release formulations, as summarized elsewhere [38,40,60,96,132]. Although statistically demonstrable improvements of total sleep time and sleep quality had been reported, complete sleep continuation over the entire night had usually not been observed. The suggestion and application of higher doses (50 or 100 mg) for further improving sleep maintenance [97,98] appeared reasonable and was partially successful. However, even highest doses such as 300 mg enterally did not warrant full sleep maintenance in a substantial number of subjects in a treated cohort [99]. In other words, melatonin is not just a sleeping pill. The pathophysiological causes of insomnia matter for all the attempts of improving sleep. This reservation has to be made for the synthetic melatonergic agonists, too. 

However, higher doses of melatonin (up to 600 mg, or respective amounts in animals according to dose translation) have been successfully applied, especially for purposes of antioxidative and anti-inflammatory treatment, including rescuing from ischemia, viral or bacterial infections and sepsis [86,100,105,133,134,135,136,137,138,139,140,141,142,143]. The aim of protecting lives against acute potentially deadly pathologies fully justify the use of high doses, regardless of whether the treatment may disturb the circadian system. However, perturbations of clocks may not be as severe as one might think as long as melatonin is only regarded as a single component of the circadian system. There is no good reason for believing that oscillators would be stopped by high melatonin, i.e., a compound that shifts clocks or extends periods by only a very few hours. Melatonin’s additional advantage of detoxifying, at elevates dosage, highly reactive and damaging intermediates should not be underrated, a property that is not shared by synthetic melatonergic drugs. Moreover, the supreme tolerability of melatonin must be appreciated.
